# Knockdown of RNF6 inhibits HeLa cervical cancer cell growth via suppression of MAPK/ERK signaling

**DOI:** 10.1002/2211-5463.13216

**Published:** 2021-06-18

**Authors:** Kang Zhu, He Bai, Mingzhu Mu, Yuanyuan Xue, Zhao Duan

**Affiliations:** ^1^ Department of Obstetrics and Gynecology The Second Affiliated Hospital Xi'an Jiaotong University China; ^2^ Department of General Surgery Department The First Affiliated Hospital of Xi’an Medical University China

**Keywords:** cell growth, cervical cancer, ERK, RNF6

## Abstract

Ring finger protein 6 (RNF6) is implicated in various human malignancies, but its function in cervical cancer (CC) is incompletely understood. Here, we explored the biological significance of RNF6 in HeLa CC cells and the underlying regulatory mechanisms. The expression of RNF6 was observed to be high in both primary tissues and CC cells. RNF6 promoted HeLa CC cell growth. Knockdown of RNF6 in CC cells resulted in suppression of proliferation and promotion of apoptosis. Moreover, elevation of RNF6 had an adverse effect on the prognosis of CC. Subsequent analyses showed that these effects may be mediated via activation of ERK signaling. These findings provide evidence that the knockdown of RNF6 suppresses the MAPK/ERK pathway to regulate the growth of CC cells, which suggests that RNF6 may have potential as a target for diagnosis and treatment for CC.

## Graphical abstract

We confirmed that ring finger protein 6 (RNF6) expression was significantly increased in cervical cancer (CC) tissues and HeLa cells, and this was associated with poor prognosis of CC patients. Knockdown of RNF6 inhibited cell proliferation and promoted apoptosis by suppression of the MAPK/ERK signaling pathway in HeLa cells. Consequently, targeting the RNF6‐ERK pathway may have potential for the development of treatments for CC.

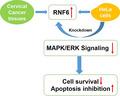

**Abbreviations**
AR: androgen receptor
CC: cervical cancer
CIN: cervical intraepithelial neoplasia
ERK: extracellular signal‐regulated kinase
GEPIA: Gene Expression Profiling Interactive Analysis
IHC: Immunohistochemical
MAPK: mitogen‐activated protein kinase
qRT‐PCR: quantitative real‐time polymerase chain reaction
RNF6: ring finger protein 6
siRNA: small interfering RNA
TCGA: The Cancer Genome Atlas
UPP: ubiquitin–proteasome pathway

## Background

Cervical cancer (CC) is the most common gynecological malignant tumor and seriously endangers the health and lives of women in developing countries. Common treatments for CC include surgery and chemotherapy, radiotherapy, or both. However, patients with advanced CC still have a poor prognosis, which requires early diagnosis and prediction to greatly improve the treatment outcome. Therefore, further studies are needed to develop novel therapeutic approaches for CC.

Ring finger protein 6 (RNF6) is an E3 ligase [1], and its role in tumorigenesis has been debated extensively. Mutated RNF6, which is located on chromosome 13q12, was first found to regulate human esophageal squamous cell carcinoma as a tumor suppressor [2]. More recently, its role in carcinogenesis is emerging as cancer research progresses.

RNF6 exerts different effects in various cancers and can be used as a tumor‐specific target. A recent study confirmed that RNF6 is an oncogene for gastric cancer [3]. That study proposed a promising role of RNF6‐SHP‐1‐STAT3 in gastric cancer cell growth. Many studies have recently shown that high expression of RNF6 indicates a poor prognosis of colorectal cancer as an independent factor. RNF6 is overexpressed in colorectal tumors and activates JAK/STAT3 or Wnt/β‐catenin pathways to promote colorectal tumorigenesis [4]. Patients with cisplatin‐resistant lung adenocarcinoma have increased expression of RNF6 in tumor cells [5]. RNF6 atypically polyubiquitinates at Lys‐6 and Lys‐277 and facilitates transcriptional activity of the androgen receptor (AR) because of its overexpression in prostate cancer. By regulating AR functions, RNF6 promotes prostate cancer growth. Mutations in the RNF6 gene and specific inhibition change AR transcriptional activity in xenograft models and delay the progression of prostate cancer [6]. Transcription factor PBX1 directly targets RNF6, and increased levels of RNF6 expression in leukemic cells promote their growth [7]. However, there are few studies on RNF6 and limited information about its biological functions. The role of RNF6 in cervical malignancies has yet to be determined.

In this study, we measured the expression of RNF6 in CC cells and assessed its possible molecular mechanisms in cell growth and cell apoptosis. We found that CC cells proliferated significantly because of increased expression of RNF6. Moreover, RNF6 might function via activation of the mitogen‐activated protein kinase/extracellular signal‐regulated kinase (MAPK/ERK) pathway.

## Methods

### Cells and tissues

The HeLa CC cell line was provided by the Key Laboratory of Environment and Disease, Medical College of Xi’an Jiaotong University. The cells were authenticated and tested for mycoplasma. Cells were cultured in DMEM with 10% FBS and 1% penicillin/streptomycin in a standard CO_2_ (5%) cell incubator at 37 °C.

A total of 40 pathologically confirmed specimens were surgically resected or biopsied at the Department of Pathology, the Second Affiliated Hospital of Xi’an Jiaotong University, China. The cases included 20 paraffin‐embedded cervical squamous cell carcinoma tissue blocks and 20 adjacent nontumor tissue blocks. The patients had not received any other treatment before surgery, such as chemotherapy or radiotherapy.

All samples were obtained with written informed consent under a protocol conformed to the guidelines set by the Declaration of Helsinki and approved by the Ethics Committee of the Medical School of Xi'an Jiaotong University. The study was compliant with all relevant ethical regulations regarding research with human participants.

Ethical approval was obtained from the Ethics Committee of the Medical School of Xi’an Jiaotong University. All samples were obtained in accordance with the relevant ethical regulations.

### Plasmid construction and transfection

Two effective small interfering RNA (siRNA) sequences were designed and validated to knockdown endogenous RNF6 expression (Gima, Shanghai, China). These sequences were as follows: human RNF6‐1(siRNF6‐1): sense 5′‐CCCGAACAAUGGAGAGUUUTT‐3′; antisense 5′‐AAACUCUCCAUUGUUCGGGTT‐3′; human RNF6‐2(siRNF6‐2): sense 5′‐GGAUCCGUCCUGGAGAAAATT‐3′; and antisense 5′‐UUUUCUCCAGGACGGAUCCTT‐3′; negative scramble control sequences(si‐NC) were as follows: sense 5′‐UUCUCCGAACGUGUCACGUTT‐3′ and antisense 5′‐ACGUGACACGUUCGGAGAATT‐3′; and an RNF6 overexpression plasmid was designed to force expression of RNF6 in HeLa CC cells. Lipofectamine 3000 reagent (Invitrogen, Carlsbad, CA, USA) in DMEM (Gibco, Carlsbad, CA, USA) was used in accordance with the manufacturer’s instructions. The overexpression plasmid map is shown in Fig. S1. In the RNF6+PD98059 group, HeLa cells were treated with 50 μm ERK1/2 blocker PD98059 (CST, Beverly, MA, USA) after transfection of the RNF6 overexpression plasmid [8].

### Quantitative real‐time reverse transcription‐PCR

Cells were harvested at 24 h after transfection. Total RNA was extracted using TRIzol reagent, chloroform, and isopropanol (Invitrogen, USA) in accordance with the manufacturer’s protocols. cDNA was reverse transcribed by a PrimeScript™ RT Reagent Kit (GeneStar). Quantitative real‐time polymerase chain reaction (qRT‐PCR) was performed to measure the mRNA level of RNF6 using a SYBR Supermix Kit (Bio‐Rad Laboratories, Richmond, CA, USA). The thermocycling conditions were 40 cycles of 10 min at 95 °C, 1 min at 60 °C, and 30 s at 72 °C. β‐Actin was used as an internal reference. The primers used were as follows: RNF6, forward 5′‐AGAAGATGGCAGCAAGAGCG‐3′ and reverse 5′‐TCAAGTCAGGCTGAGATGCTAGT‐3′; β‐actin forward, 5'‐ATCTGGCACCACACCTTCTA‐3' and reverse, 5'‐GGATAGCACAGCCTGGATAC‐3'.

### MTT assay

Cell proliferation was analyzed by a 3‐(4,5‐dimethylthiazol‐2‐yl)‐2,5‐diphenyl tetrazolium bromide (MTT) assay (Sigma, USA). After transfection for 24, 48, and 72 h, cells were harvested and seeded at 3500 cells per well in 100 μL culture medium in a 96‐well culture plate. MTT (10 μL per well, 5 mg·mL^−1^) and dimethyl sulfoxide (DMSO; 150 μL per well) were added to the plates, and cell proliferation was assessed using a microplate reader. The optical density of the wells was measured at 492 nm [8, 9]. Each experiment was repeated three times.

### Immunohistochemical staining

Immunohistochemical (IHC) staining was performed to measure the expression of RNF6 protein in CC tissue samples. In brief, tissues were embedded in wax after soaking in 4% paraformaldehyde, ethanol, and xylene. DAB was used for counterstaining. Tissue sections were dehydrated and mounted. Finally, the sections were viewed under a microscope in brightfield. The staining intensity was assigned as follows: 0 (negative), 1 (weak), 2 (moderate), and 3 (strong). The percentage of nucleus‐positive cells was scored as follows: 1 (0%–25%), 2 (26%–50%), 3 (51%–75%), and 4 (76%–100%). RNF6 expression was initially defined as the immunoreactive score (IRS) [10], the product of these two, and IRS was represented by three categories: weak (0–3), moderate (4–6), and strong (8–12).

### Western blot analysis

After transfection for 24 h, cells were harvested and lysed in RIPA buffer (Beyotime, Shanghai, China). Equivalent amounts of proteins were determined using a BCA protein assay kit (Biyuntian Biotech, Shanghai, China). Protein samples were subjected to 10% sodium dodecyl sulfate/polyacrylamide gel electrophoresis and then transferred to a PDVF membrane (Millipore, Billerica, MA, USA). The blot was incubated with the primary antibody overnight at 4 °C. The primary antibodies used in this study were as follows: anti‐RNF6 (1 : 1000 dilution; ab204506, Abcam, Cambridge, MA, USA), anti‐ERK1/2 (1 : 1000 dilution; 11257‐1‐AP, ProteinTech Group, Chicago, IL, USA), anti‐p‐ERK1/2 (1 : 1000 dilution; 4376S, Cell Signaling Technology, Inc., Beverly, MA, USA), anticaspase‐3 (1 : 1000 dilution; 9662S, Cell Signaling Technology, Inc.), anticleaved caspase‐3(1 : 500 dilution; 9661S, Cell Signaling Technology, Inc.), and anti‐GAPDH (1:3000 dilution) (5174S, Cell Signaling Technology, Inc,). Blots were then incubated with a horseradish peroxidase‐conjugated goat anti‐rabbit secondary antibody (1:5000 dilution) (7074S, Cell Signaling Technology, Inc., Beverly, MA, USA) for 1 h at 37 °C. Signals were detected using enhanced chemiluminescence reagents (Pierce, USA). Quantity One software (Bio‐Rad, USA) was used to analyze the intensity of protein bands in blots. GAPDH was used as the loading control.

### Apoptosis assay

An apoptosis assay was performed by flow cytometry with Annexin V‐FITC/propidium iodide (PI) staining. After transfection for 24 h, cells were harvested and seeded at 1 × 10^5^ cells per well in 100 μL culture medium in a 12‐well culture plate. The cells were washed twice with PBS and resuspended in 400 μL Annexin V‐FITC binding buffer. Then, 5 μL Annexin V‐FITC and 10 μL PI were added to stain the cells in accordance with the instructions of the Apoptosis Detection Kit (Qihan Biotech, Shanghai, China). Then, apoptosis was detected on a flow cytometer (BD Biosciences, USA). The experiment was repeated three times.

### Database analysis

The Gene Expression Profiling Interactive Analysis (GEPIA) database was developed by Peking University. On the basis of information in the Cancer Genome Atlas (TCGA) and Genotype‐Tissue Expression Projects, differential expression of genes in tumors and normal tissues was analyzed by a single gene, multiple genes, and tumor types. GEPIA can be directly linked to other large databases or literature libraries such as GeneCard, PubMed, COSMIC, and HPA.

### Statistical analysis

All data were analyzed for statistical significance using spss 22.0 software (SPSS Inc., Chicago, IL, USA). Student’s *t*‐test was used for comparisons between two groups, and one‐way analysis of variance (ANOVA) was used for comparisons among multiple groups. A value of *P* < 0.05 was considered to indicate a statistically significant difference.

## Results

### RNF6 is overexpressed in CC

**Fig. 1:**
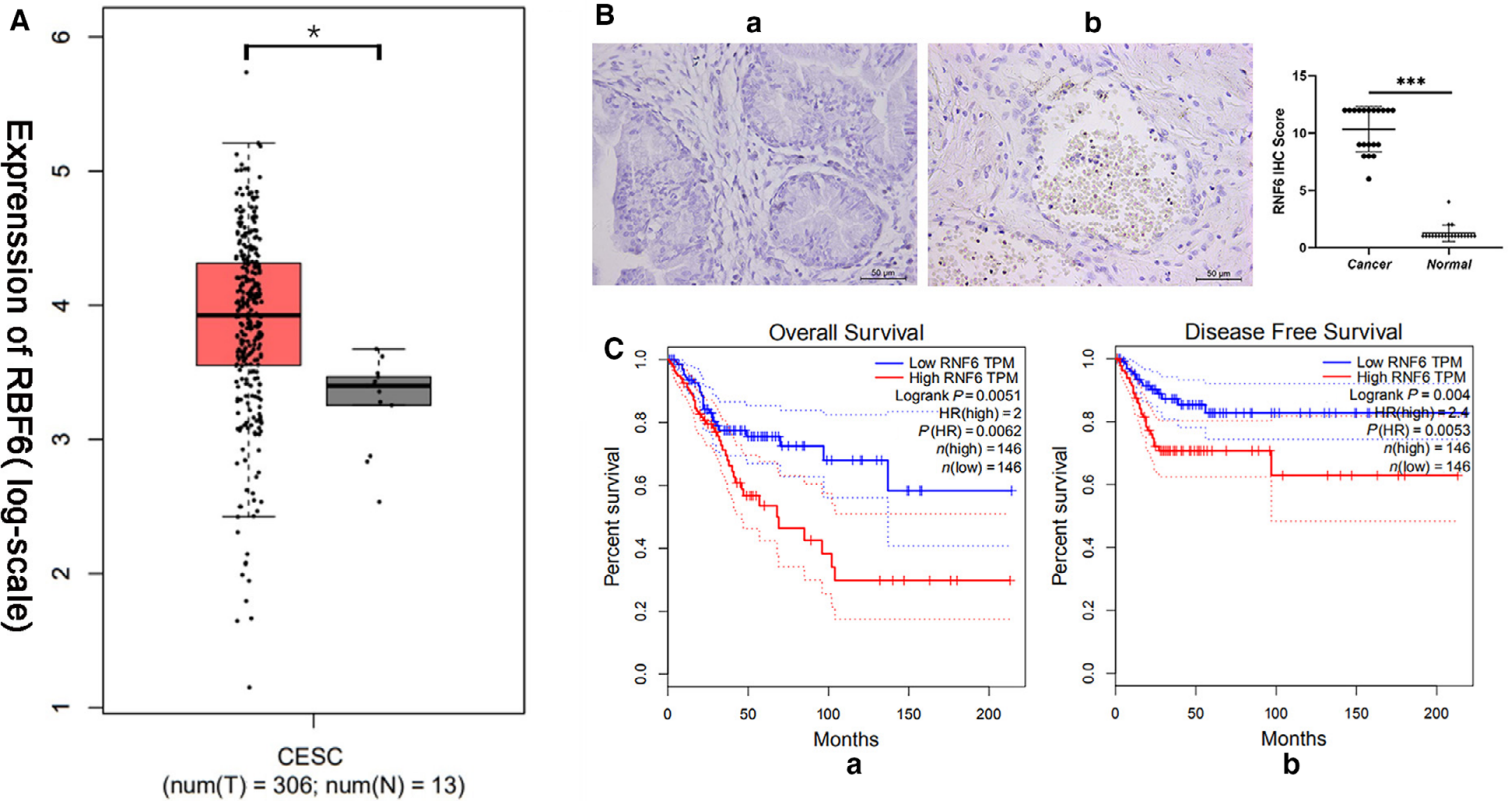
RNF6 is overexpressed in CC. (A) GEPIA was used to analyze the expression level of RNF6. T, tumor tissue; N, normal tissue. (Mean ± SD; *n* = 20; Student’s *t*‐test). *P < 0.05 (B) IHC staining of the expression of RNF6 in CC tissues and adjacent nontumor tissue. Scale bar = 50 μm (a) no brown or yellow particles in nontumor tissues; (b) a large number of brown‐yellow particles in CC tissues. (C) GEPIA was used to investigate the prognostic significance of RNF6 expression in CC patients. (a) RNF6 overexpression and its positive correlation with poorer overall survival in CC patients. (b) RNF6 overexpression and its positive correlation with poorer disease‐free survival in CC patients.

To evaluate the expression level of RNF6 in CC, GEPIA was first used for analysis. The expression of RNF6 in CC tissues was significantly increased (Fig. 1A). Next, we collected 20 pairs of adjacent normal tissues and tumor tissues from CC patients to measure the protein level of RNF6 that was also overexpressed in tumor tissues (Fig. 1B). Moreover, we investigated the prognostic significance of RNF6 expression in CC patients using GEPIA with the log‐rank test (Fig. 1C). We found that high expression of RNF6 indicated a poor prognosis of CC patients.

### RNF6 promotes CC cell growth

**Fig. 2:**
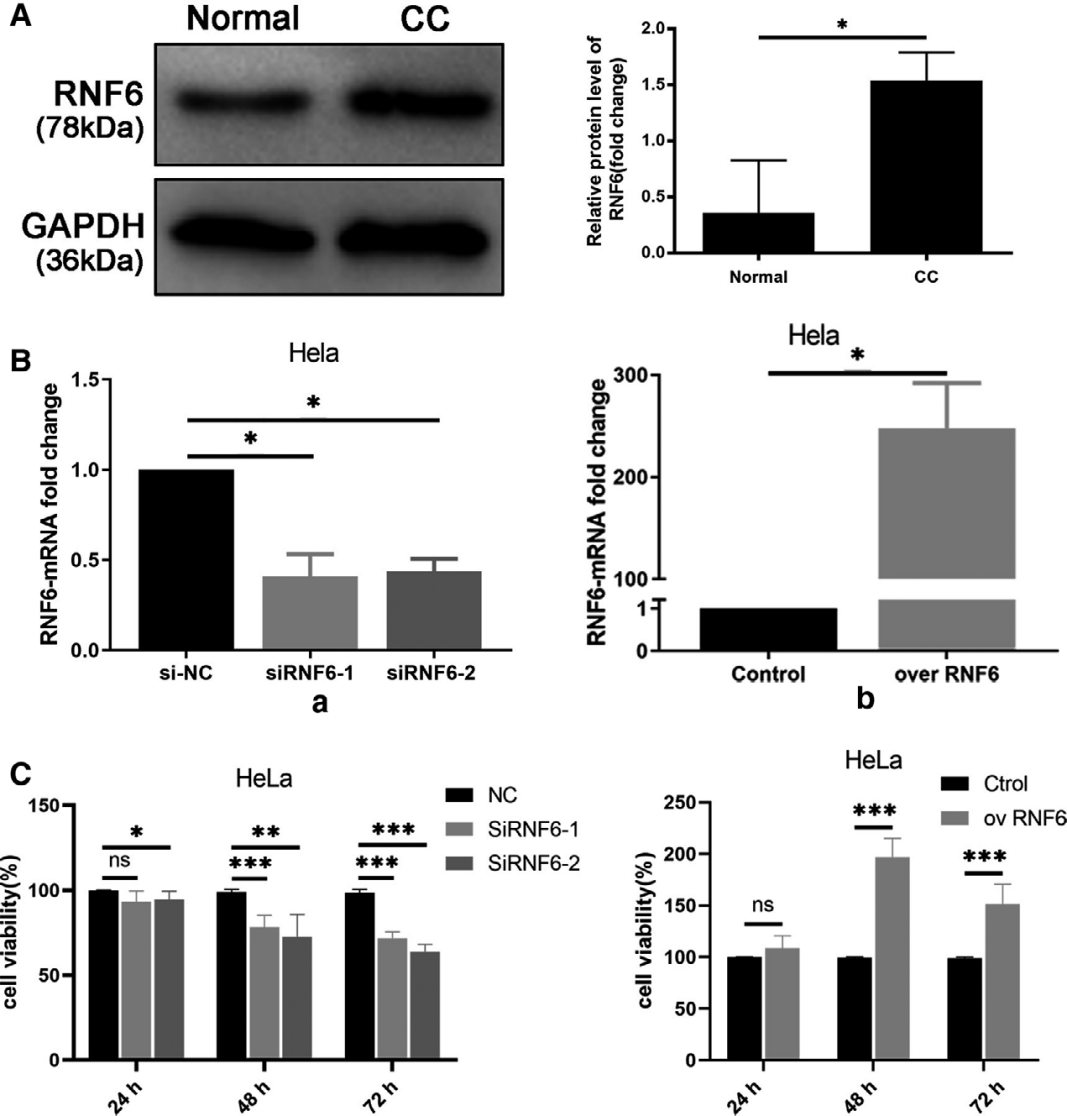
RNF6 promotes CC cell growth. (A) The protein level of RNF6 was measured by western blotting. *P < 0.05 (B) The transfection efficiency was measured by qRT‐PCR. *P < 0.05 (a) RNF6 siRNA‐1, RNF6 siRNA‐2, or si‐NC was transfected into HeLa cells. (b) RNF6 overexpression plasmid or empty plasmid was transfected into HeLa cells. (C) MTT assays was used to assess cell viability at certain points in time. (Mean ± SD; *n* = 4; Student’s *t*‐test and one‐way ANOVA). *P < 0.05.

We knocked down expression of RNR6 and assessed cell activity to determine the biological functions of RNF6. First, we examined RNF6 expression in HeLa cells by western blotting (Fig. 2A). Consistent with the tumor tissues, RNF6 expression was high in the CC cell line. To further explore the biological functions of RNF6, we validated two effective siRNA sequences to knock down endogenous RNF6 expression (Fig. 2B). We found that the proliferation of HeLa cells was significantly suppressed in MTT assays after knockdown of RNF6. Conversely, RNF6 overexpression significantly enhanced cell proliferation (Fig. 2C). Taken together, CC cell growth was promoted by RNF6 overexpression.

### RNF6 inhibits apoptosis of CC cells

**Fig. 3:**
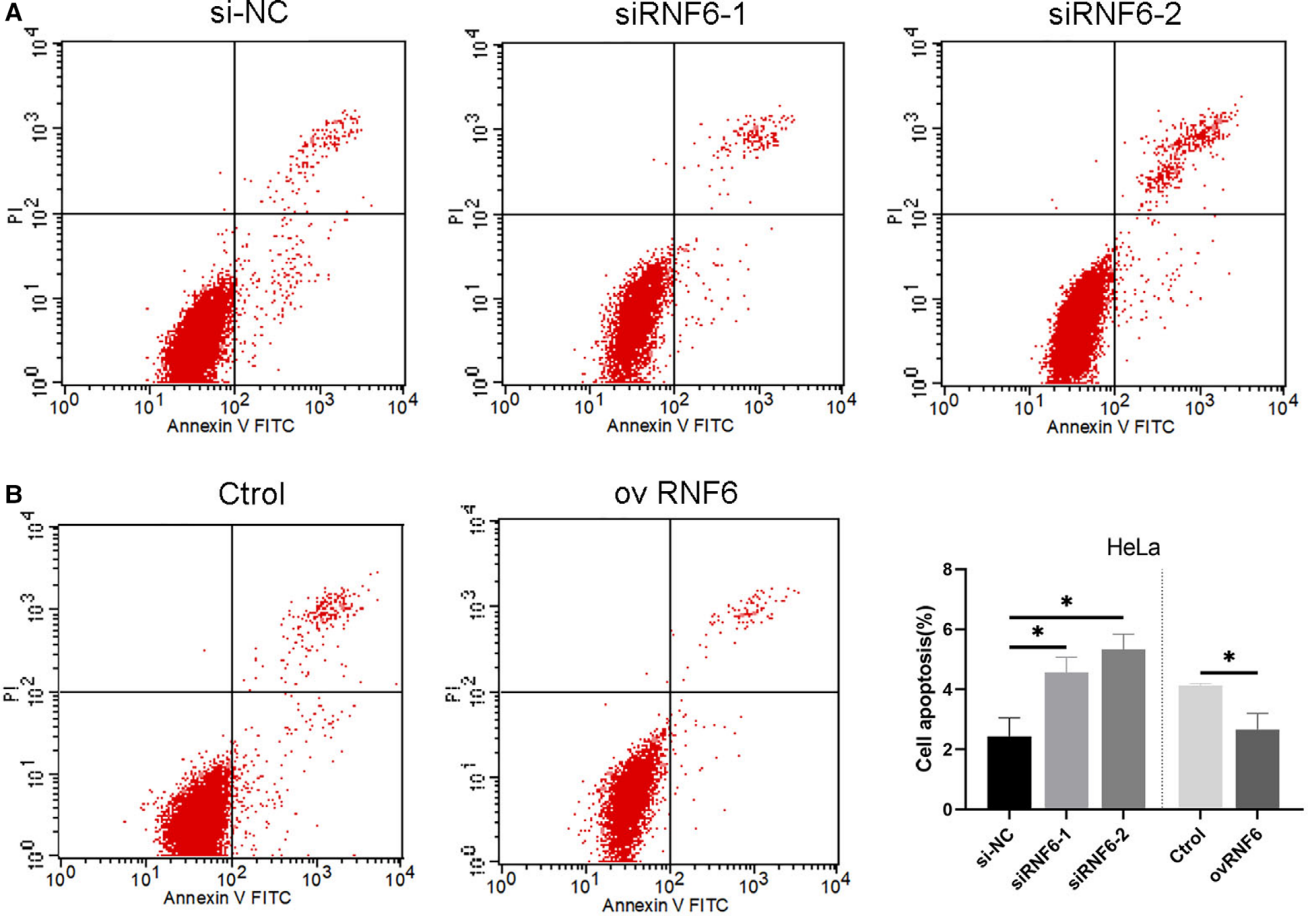
RNF6 inhibits apoptosis of CC cells. (A) Cell apoptosis was promoted after knockdown of RNF6 in HeLa cells. Quadrants: upper left (necrotic cells); lower left (live cells); lower right (early apoptotic cells); and upper right (late apoptotic cells). (B) Cell apoptosis was inhibited after RNF6 overexpression. (Mean ± SD; *n* = 4; Student’s *t*‐test; and one‐way ANOVA). *P < 0.05.

Apoptosis has been implicated in tumorigenesis and development. After transfection for 24 h, we performed flow cytometry to detect apoptosis to determine whether apoptosis was triggered by silencing RNF6. Compared with the control group, the total apoptosis ratio was significantly increased after knockdown of RNF6 (Fig. 3A). Conversely, apoptosis was significantly inhibited by forcing expression of RNF6. Additionally, the total apoptosis ratio was evidently decreased compared with empty plasmid‐transfected cells (Fig. 3B).

### RNF6 activates the MAPK/ERK pathway in CC cells

ERK1/2 has been reported to be overexpressed in CC cells [11, 12]. Additionally, abnormal activation of ERK signaling pathway inhibits multiple aspects of apoptosis and promotes tumor cell growth [13]. We hypothesized that RNF6 might also mediate biological behaviors of CC cell through the ERK pathway.

**Fig. 4:**
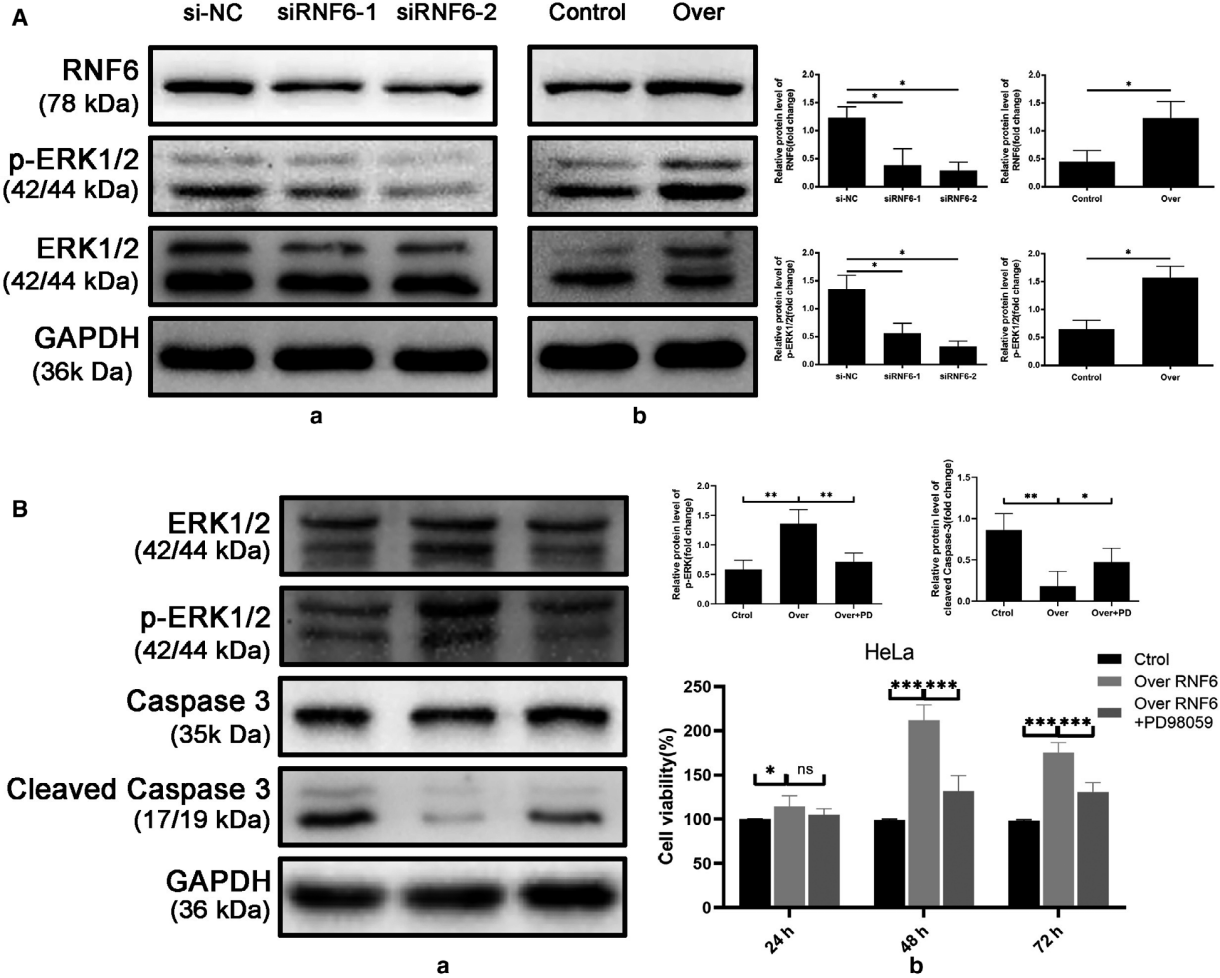
RNF6 activates the MAPK/ERK pathway in CC cells. (A) After transfection for 24 h, cells were harvested and detected by western blotting. GAPDH was used as the loading control. (a) Silencing RNF6 decreased p‐ERK1/2. (b) The expression level of p‐ERK1/2 was increased by upregulating RNF6, while total ERK1/2 expression was unaffected. (B) Western blotting for ERK1/2, p‐ERK1/2, caspase‐3, cleaved caspase‐3, (a) and MTT assays for RNF6 overexpression cells (b) treated with or without the ERK1/2 blocker PD98059. (Mean ± SD; *n* = 4; Student’s *t*‐test and one‐way ANOVA). *P < 0.05.

First, we found coexpression of RNF6 and phosphorylated ERK1/2 (p‐ERK1/2). Next, western blotting was performed as shown in Fig. 4A. Silencing RNF6 decreased p‐ERK1/2 at 24 h after transfection. Conversely, the expression levels of p‐ERK1/2 were increased by upregulating RNF6, while total ERK1/2 expression was unaffected (Fig. 4A).

To confirm whether RNF6 regulated CC progression via ERK1/2 pathway activation, we used the ERK1/2 blocker PD98059 to observe changes in the proliferation and apoptosis of HeLa cells mediated by RNF6. Cell proliferation was inhibited, and apoptosis was increased in RNF6‐overexpressing cells treated with PD98059 compared with the untreated control (Fig. 4B). These results indicated that the effects on cell proliferation and apoptosis exerted by RNF6 overexpression were significantly attenuated by blocking the MAPK/ERK pathway.

## Discussion

The presence of high‐risk human papillomavirus (HR‐HPV) types, such as 16 and 18, is a well‐established cause of cervical carcinomas [14]. Of course, only a small percentage of HPV‐infected patients develop CC and other cofactors may be involved in the development of CCs. Therefore, searching for factors other than HPV infection has become a hot spot in research of CC. Studies have shown that ubiquitin–proteasome pathway (UPP) is closely related to the occurrence and development of tumors in some female‐related diseases. Studies related to CC originated from HPV suggested that p53 is degraded through the UPP [15, 16], which leads to changes in the cell cycle and loss of apoptotic function. As an upstream component of the ubiquitin–proteasome system, E3 ligase RNF6 has attracted increasing attention in the tumor field. Growing evidence indicates overexpression of RNF6 in CC and that this can cause distant metastasis, which ultimately leads to a poor prognosis of CC patients.

However, the role of RNF6 in carcinogenesis remains unclear. The function and clinical role of RNF6 in CC have not yet been fully elucidated. In our study, RNF6 acted as an oncogene in CC. To characterize the expression pattern of RNF6, we performed immunohistochemistry in 20 CC tissues and western blotting in HeLa cells. Consistent with the data from GEPIA, RNF6 protein expression level was substantially increased compared with normal tissues, which indicated that RNF6 participated in the tumorigenesis of CC. Moreover, the data from GEPIA showed that the clinical prognosis of patients with RNF6‐positive staining was significantly poor. In accordance with the multivariate analysis, RNF6 overexpression was considered to be an independent prognostic indicator. Conversely, RNF6 was first found to be mutated on chromosome 13q12 and acted as a tumor suppressor in human esophageal squamous cell carcinoma [2]. Our study confirmed that RNF6 may exert variable or even opposing roles in different tumor contexts and may be a novel candidate for cancer immunotherapy.

Proliferation of cancer cells is a sign of a malignant tumor and poor prognosis of cancer patients. In our study, we designed two effective siRNA sequences to knock down endogenous RNF6 expression and an RNF6 overexpression plasmid to enforce RNF6 expression in HeLa CC cells to assess the effects of RNF6 and obtain a greater understanding of the potential association between the cell biological characteristics and expression levels of RNF6. In vitro experiments suggested that RNF6 inhibited apoptosis to promote cell proliferation, which was consistent with the results obtained from tumor tissues. These results indicated that RNF6 played its oncogenic role through promoting proliferation and suppressing apoptosis of CC cells.

The MAPK/ERK pathway functions in regulating various physiological and pathophysiological processes in the initiation and progression of different types of cancers [17, 18]. ERK is a crucial member that could transmit various extracellular stimulation signals into the cell [19]. Additionally, most current studies believe that abnormal activation of the ERK signaling pathway inhibits multiple aspects of apoptosis and promotes tumor cell growth [13]. Recent studies have demonstrated that RNF135 and RNF138 amplification activate the ERK signaling pathway in glioblastoma, which promotes cellular proliferation, migration, and invasion [20, 21]. ERK overexpression has been found in CC. Additionally, during development from cervical intraepithelial neoplasia (CIN) I to CIN III, the MAPK/ERK pathway was significantly activated [11]. This conclusion is consistent with the finding that the MAPK/ERK signaling pathway plays an important role in the evolution of CC and promotes the cancerous tendency of cervical cells [12]. Therefore, we explored whether high expression of RFN6 promoted cell proliferation together with its possible molecular mechanisms.

The activation of the ERK signaling pathway requires activation of ERK by phosphorylation, which mediates the transmission of signals from the cytoplasm to the nucleus by up‐ or downregulating nuclear transcription factors such as c‐myc, c‐Fos, c‐Jun, cyclin D1, and Bcl‐2 [22]. c‐Fos and c‐Jun as oncogenes that encode nuclear proteins are highly expressed in CC [23]. It has been demonstrated that ERK may affect HeLa cell proliferation by regulating the expression of c‐Fos and c‐Jun proteins [24]. In the present study, we performed western blot analysis to measure the expression of p‐ERK1/2 and total ERK1/2 after forcing expression of RNF6. While the former was notably elevated, the latter was unaltered. Additionally, we observed that cell proliferation was inhibited and apoptosis was increased in RNF6‐overexpressing cells after blocking the MAPK/ERK pathway with the ERK1/2 blocker PD98059. Therefore, we speculated that we could knock down RNF6 accompanied by inhibition of the MAPK/ERK signaling pathway as well as its downstream genes to treat CC. The intrinsic mechanisms remain unknown and require further study.

Our study had some limitations that future research should address. Because of inadequate funding, *in vitro* and animal experiments have not yet been implemented to determine the specific molecular mechanisms of RNF6‐ERK signaling. Therefore, we will conduct further experiments in future.

We confirmed that RNF6 expression was significantly increased in CC samples and HeLa cells compared with normal tissues, which indicated a poor prognosis of CC patients. Knockdown of RNF6 inhibited cell proliferation and promoted apoptosis by suppression of the MAPK/ERK signaling pathway in CC cells. Our study has provided a direction in CC research and might provide important information to better understand the mechanisms of RNF6‐related cervical carcinogenesis. Further studies of RNF6 may develop better therapeutic strategies for CC.

## Conclusion

This study showed that RFN6 acts as an oncogene to promote cell growth by activating the MAPK/ERK signaling pathway in CC cells. Consequently, targeting the RNF6‐ERK pathway may be an effective and promising treatment for CC.

## Conflict of interest

The authors declare no conflict of interest.

## Author contributions

KZ and MZM collected associated clinical data and performed the experiments. HB and YYX contributed to statistical analyses of the data. ZD planed and designed the research. KZ and HB wrote the manuscript and conceptualized the framework for this research. All authors read and approved the final manuscript. KZ and HB contributed equally to this paper.

## Supporting information


**Fig. S1.** The overexpression plasmid map.

## Data Availability

The datasets used and/or analyzed during the present study are available from the corresponding author on reasonable request.
