# Supplementary figures and images for: Knockdown of RNF6 inhibits HeLa cervical cancer cell growth via suppression of MAPK/ERK signaling
Source: FEBS Open Bio. 2021 Jun 18;11(7):2041–9. doi: 10.1002/2211-5463.13216 (PMC8255836; doi:10.1002/2211-5463.13216)

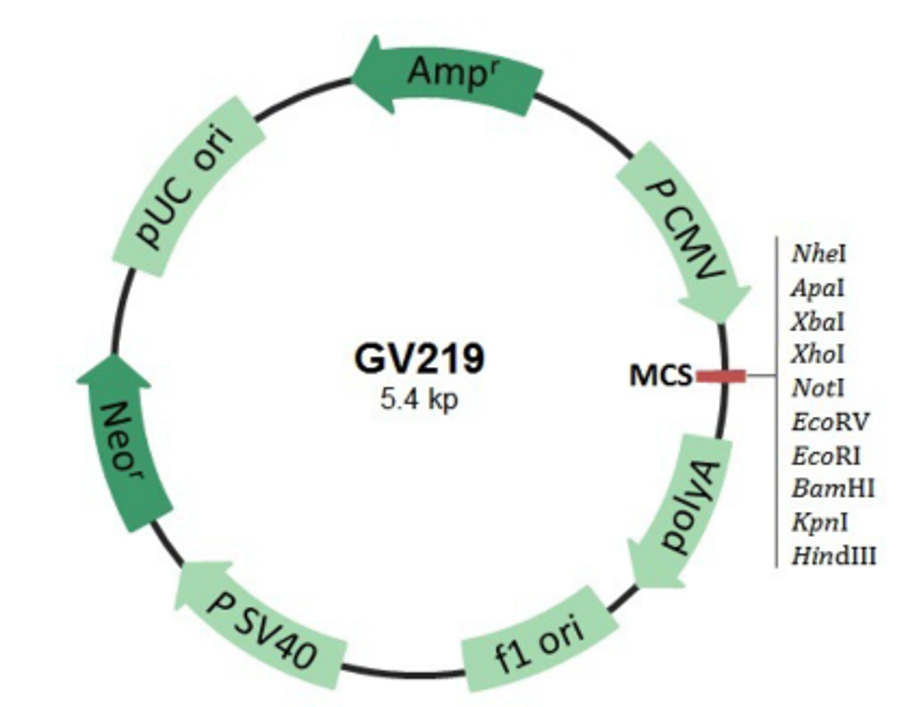

Supplement: Supplementary file 1 — Fig. S1. The overexpression plasmid map. [file FEB4-11-2041-s001.tif]
